# Measuring Perceived Discrimination and Its Consequences for Latino Health

**DOI:** 10.3390/soc15120333

**Published:** 2025-11-28

**Authors:** Giovani Burgos, Alex Trillo

**Affiliations:** 1 Department of Sociology, College of Arts and Sciences, Adelphi University, Garden City, NY 11530, USA; 2 Department of Sociology, Urban Studies & Anthropology, Saint Peter’s University, Jersey City, NJ 07306, USA;

**Keywords:** Latinos, discrimination, depression, CES-D, chronic health, racism, structural equation modeling, ethnicity

## Abstract

Research demonstrates that discrimination is detrimental to health. However, most discrimination research does not examine Latino ethnic differences and often relies on unidimensional alpha scales. Such an analytic strategy obscures ethnic differences, can mask the multidimensional nature of discrimination, inflate reliability estimates, produce attenuated or spurious relationships, and bias parameters. To address these issues, we use data from the National Latino and Asian American Study to (1) examine group differences on the Everyday Discrimination Scale (EDS), (2) conduct a confirmatory factor analysis of the EDS to assess its fit and dimensionality for each Latino ethnic group, and (3) evaluate how alternative scaling approaches affect the relationship between discrimination, depression, and chronic health conditions. Results reveal significant group differences in perceived discrimination and show that a second-order factor with two dimensions—subtle and overt discrimination—fits well across all Latino groups. The relationship between discrimination and health is stronger when discrimination is modeled as a second-order factor. These findings indicate that (1) alternative scaling approaches may be more appropriate than alpha scales, (2) more precise measurement of discrimination can better capture its impact on health, and (3) disaggregating panethnic categories such as “Latino” that is essential for understanding ethnic stratification and health.

## 1. Introduction

White supremacy continues to undermine the rights and well-being of Latino communities, manifesting in violent hate, harassment, threats, color-blind ideology, and systemic discrimination [1,2]. This is concerning considering the large body of empirical evidence showing that discriminatory experiences persistently drive racial and ethnic inequality, by limiting life chances across multiple domains, including labor market participation, arrest, prosecution, incarceration, access to housing and homeownership, voter suppression and poor health outcomes [3]. Despite these findings, the current political climate has become increasingly hostile to public discussions of race, ethnicity, and the well-being of minority populations [4]. The concurrent rise of White supremacy and the persistence of color-blind ideology—which denies the existence of discrimination and seeks to erode the gains of the civil rights movement—underscores the need for methodological rigor in research on racism and health [5]. Framed within this broader context, it is imperative that scholars and policymakers produce the most accurate assessments possible of the relationship between discrimination and health disparities.

One useful approach is the use of sociologically validated survey scales of perceived discrimination to evaluate how this stressor influences health disparities. Research shows that such questions adequately capture discriminatory experiences and are consistently linked to negative health outcomes [6]. The importance of using robust and validated scales is particularly evident in studies of Latinos, who now represent the largest minority group in the United States (U.S.) and remain especially vulnerable—though to varying degrees—to the latest wave of anti-immigrant sentiment [7]. Yet, most research on discrimination and health continues to rely on scaling approaches that risk misestimating its effects and, in turn, may inadvertently weaken arguments for advancing health equity [8].

This article addresses calls for more sophisticated measures and analyses of discrimination among Latinos along four fronts. First, we disaggregate the Latino category into Mexicans, Puerto Ricans, Cubans, and a combined category of other Latino ethnic groups. As the Latino population grows larger and more diverse, it is important to examine each group’s particular experiences because there is a relative dearth of research that examines the effects of discrimination between different Latino ethnic groups [9].

Second, drawing on Krieger et al.’s (2005:1588) still-relevant observation that more research should employ “validated multi-item self-report measures” of discrimination [10], we compare reports of perceived discrimination across Latino ethnic groups using the nine items of the Everyday Discrimination Scale [11]. To date, only a limited number of studies have applied advanced methodologies, such as structural equation modeling, to examine discrimination among individual Latino groups, and even fewer have directly contrasted group differences using this approach [12–14]. Such methods yield more reliable and valid estimates of the association between discrimination and health outcomes.

**Figure 1.**
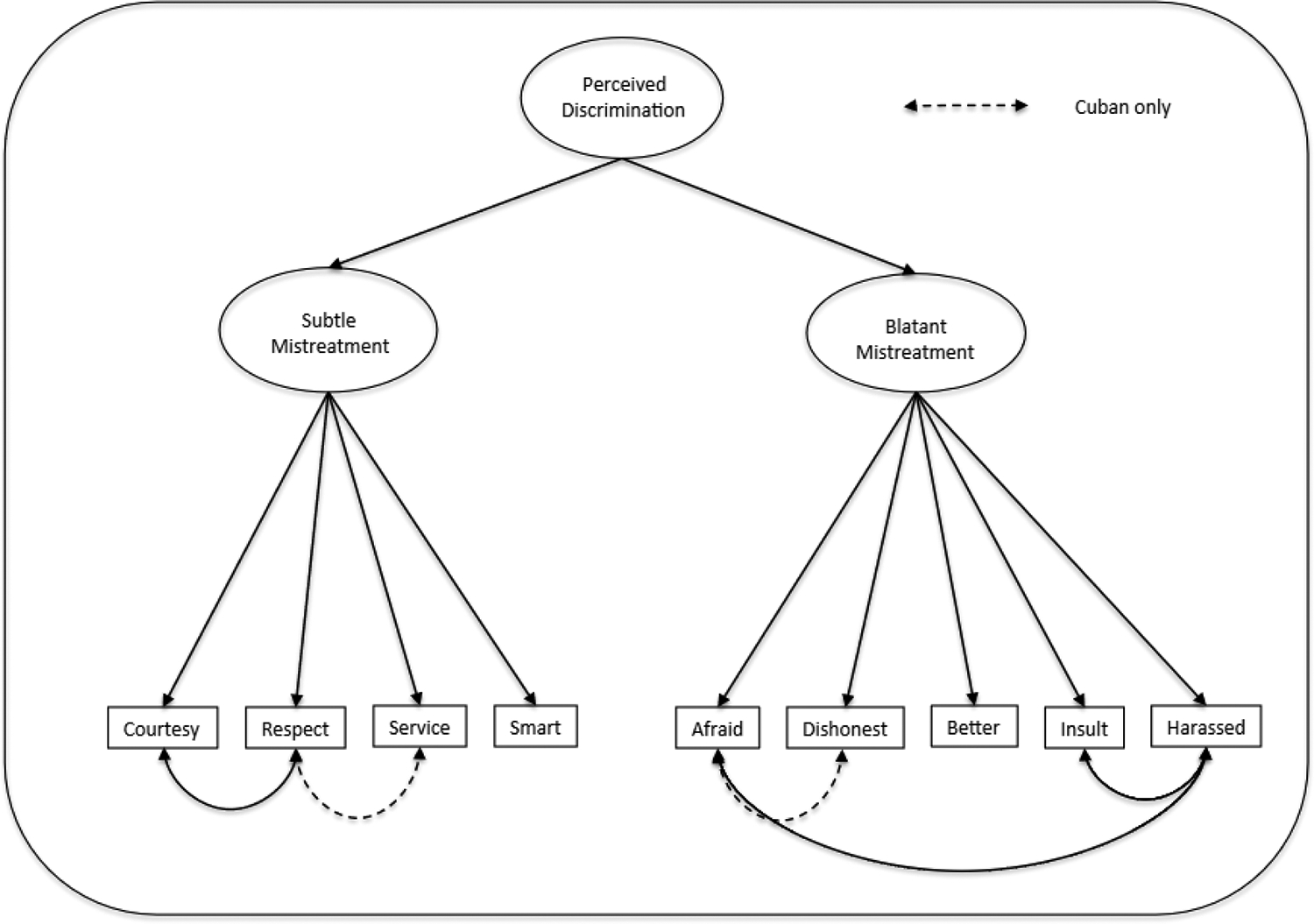
Second Order Factor of Perceived Discrimination.

Third, we evaluate the dimensional structure of the Everyday Discrimination Scale (EDS) across Latino groups using confirmatory factor analysis (CFA) and item response theory (IRT). Prior studies demonstrate that the EDS has two dimensions among African American and Asian populations [15–17], and that responses to its nine items are often highly skewed with correlated measurement error. Yet, research on Latino groups remains constrained by reliance on single-item measures or unidimensional composite (alpha) scales. These approaches neglect dimensionality, measurement error, and non-normal response distributions (i.e., ordered questions of discrimination variables), which may bias estimates of the relationship between discrimination and health outcomes [12]. To address this gap, we compare model fit across three specifications: (1) a single latent factor, (2) two correlated first-order factors (blatant and subtle discrimination), and (3) a second-order factor with two first-order dimensions (see Figure 1).

We also assess the extent to which scale fit is influenced by measurement error and by treating indicators as continuous versus categorical, drawing on IRT-based estimation [18]. This analytic strategy provides a more rigorous assessment of how perceived discrimination is structured and its potential effects on health across Latino subgroups.

Lastly, we examine how different scaling configurations of discrimination affect health outcomes for each Latino ethnic group. Recognizing the need for more accurate measurement across ethnicities, we compare multiple scaling approaches using structural equation modeling (SEM) to assess which scale most effectively captures the relationship between discrimination and health. We also evaluate the construct validity of the EDS by analyzing its association with depression and chronic physical health conditions. Specifically, we investigate whether composite scales misestimate the relationship between perceived discrimination and health when compared to multidimensional latent factor scales that account for measurement error, dimensionality, and item responses, and whether these effects vary by group. To date, sociometrically validated discrimination instruments remain relatively scarce in large-scale population health studies [19], including the NLAAS used here, which disaggregates Latino ethnic groups and examines associations between group membership and health outcomes using advanced scaling approaches.

### 1.1. Health, Discrimination Stress, and Social Stratification

Medical sociologists have long emphasized that studying health can provide valuable insights into social stratification by identifying the mechanisms (e.g., stress, social integration, status inconsistencies, SES) that link macro-level social structures to individual well-being [20]. Mental health scholars have built on this fundamental social psychological tenet on the macro-micro link to establish an impressive body of literature documenting the harmful effects that stress has on mental and physical health. This relationship is robust to the extent that individuals who experience more major life events, chronic strains, daily hassles, and other stressors are expected to have worse physical and mental health than individuals with lower stress levels in their lives [21]. Conceptually, discrimination is considered a social stressor that gives rise to distress, especially among marginalized populations such as racial and ethnic minorities [22].

Social scientists have also established that measures of perceived discrimination are empirically and theoretically appropriate to use in health survey research given that such perceptions often correspond to actual experiences with discrimination [23–25]. As Romero and Roberts (2003) note, subjective appraisals of stress, such as perceived discrimination, are sometimes better predictors of poor health than the absence or presence of stressful events [26]. This insight is also consistent with the widely accepted tenet in the stress literature that the appraisal of stressful events by individuals, and their evaluation of those events are as important in initiating stress responses as are objective indices of stress [27].

Research shows that when individuals perceive that they have been discriminated against, they tend to feel demoralized, disrespected, and most find the experience to be dehumanizing [28,29]. Harrell (2000) documents that discrimination negatively affects the life chances of individuals and can be detrimental to their psychological well-being (e.g., depression, low self-esteem, feelings of self-doubt and powerlessness, anxiety, a heightened sense of danger and vulnerability, anger, sadness), their physical health (e.g., hypertension, cardiovascular reactivity, risk behavior), their level of social integration (e.g., social connectedness and group relations), their social functioning (job performance, academic achievement, parental functioning), and their spirituality (e.g., loss of faith, meaninglessness, existential angst) [24]. More recent studies highlight the links between discrimination and chronic diseases such as hypertension and diabetes, liver disease, and asthma [30]. In short, perceived discrimination is considered a powerful source of stress since it conceptually overlaps and parallels other social stressors, including life events, chronic strains, and daily hassles [31,32].

By now there is agreement that treating discrimination under the theoretical rubric of the social stress model is analytically useful for understanding not just the health of African Americans and Asian Americans, but also the health of Latinos in the U.S. [33,34]. Research on self-reported discrimination and health reveals that while the association between perceived discrimination and physical health is inconsistent [35], the relationship between perceived discrimination and poor psychological health is more robust across different outcomes [19,36,37]. By incorporating measures of discrimination into the social stress model, health scholars have not only advanced the understanding of racial and ethnic disparities in health, but they have also elaborated on the importance of stress research in the social and medical sciences.

Nonetheless, further research is needed to understand how discrimination affects the life chances of racial and ethnic minorities. This includes developing and using reliable and valid measures of perceived discrimination among Latinos of different ethnic backgrounds. A major concern is whether current measures comprehensively and reliably capture the forms of discrimination experienced by diverse groups and whether these groups report experiences of discrimination in the same way [38]. Therefore, more research is needed to understand how these measurement issues affect the relationship between discrimination and the health of Latinos along ethnic lines.

### 1.2. Key Issues in the Measurement of Perceived Discrimination

During the past three decades, researchers have developed over 30 scales to measure experiences with racism and discrimination. These ongoing attempts to measure discrimination reflect both the complexity and importance of capturing the effects of this powerful stressor. As Meyer (2003:262) notes, the “renewed interest in prejudice as stress raises the need for theoretically based and psychometrically sound measures of prejudice,” particularly when knowledge is incomplete about the biases that may lead individuals to misreport discriminatory events [31]. However, most studies so far have been limited by poor conceptualization and measurement [6,39,40]. Researchers often rely on single-item questions or composite scales of perceived discrimination, typically constructed by adding and averaging responses to a series of questions and assessing fit with a simple check of internal consistency (i.e., Cronbach’s alpha) or by dichotomizing (1 = yes, 0 = 0) responses to multiple questions. These methods fail to capture a range of discriminatory experiences consistent with other measures of perceived stress under the social stress model.

The single-item or question approach does not capture subtle forms of discrimination, which may be more strongly related to poor health than blatant types of discrimination. For instance, a question that asks whether a person has been insulted or harassed (a blatant type of discrimination) may not capture subtle forms of discrimination (e.g., people thinking the respondent is not smart) that may be more strongly related to poor health [17]. As a result, relying on single measures of discrimination can misestimate (e.g., attenuate) the relationship between discrimination and health [41].

Similarly, the alpha scaling approach may not be the best analytic choice, especially when the underlying assumptions of this technique are violated [42,43]. For example, two key assumptions of alpha scales are that the scale items are free of measurement error, and that the errors of the scale items are independent or not correlated with each other [44]. In practice, it is impossible to measure behavioral outcomes without error. In general, these assumptions may not be reasonable in survey-based questions on subjective perceptions, which do not happen in isolation from each other and tend to be systemically linked [45]. By now, it has been established in the structural equation literature that failure to account for measurement errors in the variables of a scale can bias the goodness-of-fit measures of a scale, as well as the regression parameters of the model [46].

To address these issues, researchers have leveraged structural equation models that estimate and model measurement error biases inherent in studying the effect of perceived discrimination on health. For example, one of these studies revealed a latent factor of racist discrimination with three indicators (Appraised Racist Events, Lifetime Racist Events, and Recent Racist Events) that had a much better fit than the model without correction for measurement error [47]. Another study found their model fit much better when they included a latent variable of perceived discrimination and corrected for measurement error, establishing that racist discrimination was a more powerful predictor of psychiatric symptoms than social demographic variables once they corrected for measurement error [48]. These studies suggest that survey researchers should correct for measurement error when constructing indices of perceived discrimination. To date, it remains unclear how correlated measurement error among factor indicators affects the fit of the widely used EDS among Latinos of different ethnicities, and no study, to the best of our knowledge, has examined whether measurement error biases the relationship between perceived discrimination and health among Latinos from different ethnic backgrounds.

Alpha scales have a limitation in that their reliability is only equal to their internal consistency when the underlying factor is homogeneous and one-dimensional. When the underlying factor is multi-dimensional but constructed to have only one dimension, the reliability and validity of composite alpha scales are affected [49,50]. Survey researchers and those conducting qualitative work have argued that perceived discrimination is a complex construct with multiple dimensions [48,51–53]. For example, early on, Adams and Dressler discovered a three-factor structure of discrimination, which they termed feelings in response to discriminatory acts by white persons in service occupations, neglect of the community on the part of city officials, and the adequacy of the school system and policy in serving the community, respectively [45]. They concluded that the assumption that discrimination is perceived equally or felt the same by all members of the Black community is untenable as revealed by their three-factor structure of discrimination.

Other studies also reveal the multidimensional structure of perceived discrimination across different populations and with various scales. Although Shariff-Marco et al. (2011) found that the EDS had one dimension among Latinos in California [38], they did not report the fit of the EDS among Latinos of different ethnic backgrounds. Still, a handful of studies have found the EDS to be multidimensional. Guyll et al. (2001) discovered two dimensions in the EDS [16]. The first dimension (Subtle Mistreatment) that emerged from their principal-components factor analysis consisted of five items (being treated with less respect and courtesy, being treated as not being smart by other people, people acting as if they are better than the respondent, and receiving poor service in restaurants and stores). The second dimension reported by Guyll et al. (i.e., Blatant Mistreatment) consisted of four items: being threatened or harassed, being called names or insulted, people acting as if the respondent was dishonest, and people acting as if they were afraid of the respondent. Barnes et al. (2012) also found two dimensions of discrimination in a sample of older African and White Americans, which they labeled Unfair Treatment (the following items loaded highly on this first dimension: courtesy, respect, service, better) and Personal Rejection (i.e., dishonest, afraid, insulted, harassed, smart) [54]. Moreover, among Korean immigrants in Toronto, Noh et al. (2007) found two subscales (Overt and Subtle) based on survey questions almost identical to those found in the EDS [17].

The latest research findings additionally demonstrate that perceived discrimination scales often reflect multiple dimensions, not a single undifferentiated construct. In a Portuguese community sample, Seabra et al. (2024) performed a confirmatory factor analysis and found the EDS to fit better when it was measured as a second-order factor with two dimensions [55]. Kazmierski et al. (2023) developed and validated a multidimensional discrimination–distress scale via factor analyses in a diverse sample of mother–daughter dyads and found separate dimensions of discrimination linked to race/ethnicity, gender, and class [56]. Similarly, Harnois’ (2022) analyses also revealed that respondents interpret the EDS through at least three frameworks—negative interpersonal interactions, social-inequality experiences, and racism-specific events—suggesting the scale taps multiple underlying dimensions [57].

Overall, strong theoretical and empirical support exists for the claim that perceived discrimination is a complex and multidimensional latent factor with substantial measurement error. However, studies on Latinos have not fully addressed these measurement issues, and how measurement error, question item skewness, and scale dimensionality affect the relationship between discrimination and health among Latinos of various ethnic backgrounds. In short, the dimensional structure of discrimination scales remains unknown for Latinos of different backgrounds.

### 1.3. What Are Latinos and Their Experiences with Discrimination

What ethnic groups does the Latino term encompass and what are the key research findings on the relationship between discrimination and health in this population? In this section, we first provide a brief description of what ethnic groups fall under the panethnic group Latino, with a focus on the groups found in our data. We also include an overview of the main findings on the relationship between discrimination and health among Latinos. Given space limitations, we focus our overview of Latino groups to Mexicans, Puerto Ricans, and Cubans. For more details discussions of settlement patterns of more Latino groups see the following references [58,59]. We show that Latinos have diverse migration histories and socioeconomic profiles, and as such, it is essential to control for these factors when analyzing the relationship between discrimination and health in multivariate statistical models.

The terms Latino and Hispanic are often used interchangeably in reference to individuals living in the U.S. who trace their roots to Spanish-speaking countries in Latin America and the Caribbean, including the U.S. colony of Puerto Rico. In the U.S. demographic and sociological studies, this panethnic term typically excludes Spaniards and individuals and groups from non-Spanish-speaking nations, including Brazil and Haiti [58,60]. While these groups share their roots in Spanish and European colonialism, the destruction of native people’s culture, and the African slave trade, each group has a unique history and relationship to the U.S. that can affect their vulnerability and responses to discrimination [61,62]. Therefore, it is also important to disaggregate into ethnic groups when examining the effects of discrimination on health, as Latinos are not a monolithic group.

Mexican Americans are the largest Latino group in population size [63], and before the American Invasion War lived in different Census regions throughout the U.S, including the Western (California, Nevada, Arizona, Utah, New Mexico), Mountain (Colorado, Wyoming), Midwestern (Kansas), and Southern (Oklahoma and Texas) areas. Under the Treaty of Guadalupe Hidalgo in the year 1848, Mexican nationals who remained in the conquered territories were forcefully incorporated into the Union of the U.S., thereby becoming U.S. citizens immediately [64]. Throughout the 20th century, many more migrated from Northern Mexico to the U.S., often as part of temporary worker programs. When their work was completed, they returned home to their families. This cyclical pattern had many individuals building family and community ties throughout the Western U.S. More recently, migrants from Southern Mexico have begun to settle across the rest of the nation. The newcomers tend to be phenotypically darker, indigenous-appearing, and start with minimal education. These characteristics put them at high risk of experiencing discrimination [3]. Mexican presence in the U.S. is thus characterized by multiple generations of citizens and non-citizens, with increasing diversity in appearance and socioeconomic status. Most are in the West, but the population is growing in the Northeastern, Midwestern, and Southern regions [65]. In terms of SES, Mexican Americans tend to have a higher median household income than Cubans and Puerto Ricans, yet they also share a high poverty rate with Puerto Ricans and are less likely than either group to hold a bachelor’s degree [66].

Puerto Rico became a U.S. colony in 1898 as a spoil of the war with Spain and a commonwealth territory in 1952 [61]. Unlike Mexicans and Cubans, Puerto Ricans are born with U.S. citizenship, though residents of the island do not enjoy all U.S. rights, such as voting in presidential elections [67]. However, Puerto Ricans living in the U.S. have full voting rights after meeting the criteria for state-specific voting rules. In the 1940s, large numbers of Puerto Ricans began migrating and established significant communities in NYC, Chicago, Boston, Hartford, and Philadelphia. They primarily worked in low-wage, labor-intensive jobs—including manufacturing, domestic service, agriculture, and service-sector roles. These jobs reflected both racialized labor demand and limited access to unionized or skilled employment. New York continues to have the largest Puerto Rican Population, but Orlando has become an equally large enclave, and increasingly Puerto Ricans live across most states [68]. And while many have experienced upward mobility and moved into the surrounding suburbs of poor inner-city neighborhoods that are rapidly gentrifying, Puerto Ricans also endured the core of U.S. economic restructuring and urban decline in the 1970s and 1980s. Hence, a portion became part of what scholars referred to as the urban underclass [69]. While Mexicans have large numbers of indigenous people, Puerto Ricans range from very White in appearance, to very Black. For some Puerto Ricans, geography, lower SES, and black phenotype leave them particularly vulnerable to discrimination [3]. Compared to Mexican Americans, Puerto Ricans tend to have a lower median income, a similar poverty rate, more education than Mexicans, but less education than Cubans [66], and are more likely than Mexicans to be second- or third-generation U.S. residents and to speak English fluently [70].

Cuban presence in the U.S. began mainly with the Tampa Bay cigar factories in the early 1900s, followed by a mass migration after the Revolution of 1959, when middle and upper-middle-class Cubans fled to Miami [71]. The Cubans were welcomed with a variety of social support programs to help them settle, given that the U.S. wanted to win the symbolic war proving that American Capitalism was friendlier than Soviet Communism [72]. In the late 1960s, more working-class Cubans migrated to Union City, N.J., and Chicago [73]. Most of them were White in appearance, had more education than the average Puerto Rican or Mexican migrant, and found an easy path to citizenship. The U.S. Government also aided in their migration by helping Cubans transfer their educational credentials to the U.S. and by providing support in opening businesses [74]. In 1980 and then in 1994, a more demographically diverse population of Cubans began to migrate. This newer wave of Cubans is more phenotypically black and poorer than the first wave that migrated during the Cold War. While the older first-wave migration Cubans were not always completely welcoming to second and third-wave Cuban immigrants, the new arrivals have benefited from the established enclaves, which can be buffers to discrimination. On average, Cubans are more likely to have graduated from high school and college than Mexicans and Puerto Ricans. They have a slightly smaller median household income than Mexicans, have a much lower poverty rate than both Mexicans and Puerto Ricans, and Cubans also tend to be older than both groups [66]. When older people retire, they have lower income.

Latinos’ experiences with discrimination are varied and lumping Latinos together under one pan-ethnic group obscures the relationship between discrimination and health. For instance, early on, Burgos and Rivera found that Black Latinos reported higher levels of discrimination than non-Black Latinos and that the effects of discrimination on depression were stronger for Black Latinos [75]. In a more recent nationwide study, Findling et al. (2019) found that Latino reports of discrimination are on the uptick, while a small handful of studies find differences in reports of discrimination across ethnic groups [76]. Arellano-Morales et al. (2015) used The Brief Perceived Ethnic Discrimination Questionnaire-Community Version and factor analysis to establish that Cubans are less likely to report discrimination than Mexicans and Puerto Ricans, though these effects mostly dissipate when controlling for sociodemographic factors such as income, education, acculturation, and historical context of each group’s migration [12]. Likewise, Cano et al. (2021) used a modified version of the Experiences of Discrimination (EOD) scale and found that reports of perceived discrimination are moderated by language proficiency, and differently for each ethnic group [77]. Colombians, with high English proficiency are more likely to report experiences with discrimination than Cubans, perhaps because Cubans are shielded by a large enclave. High Spanish proficiency appears to render all ethnic groups equal in terms of reporting discrimination, while low Spanish proficiency leads Mexicans to report more experiences with discrimination than other groups. Corroborating these insights, Marrow et al. (2022) found that darker skin tone resulted in more reports of discrimination for Mexican Americans in Atlanta and Philadelphia [78], and Vargas et al. (2021) show that Black Latinos report more experience of discrimination than White Latinos [3]. Taken as a whole, these studies establish that Latinos, as racialized minorities, are targets of discrimination and that experiences with discrimination vary by ethnicity, race, and their social conditions.

### 1.4. Overview and Gaps in the Literature: The Effects of Discrimination on Health

Over the past decade, a growing and large body of research has examined the risks discrimination poses to the well-being of Latinos [78,79]. Systematic reviews consistently demonstrate that discrimination adversely affects Latino health, particularly mental health outcomes [80,81]. Moreover, studies reveal that the impact of discrimination on health is often mediated or moderated by various social factors, including social support, stress, immigrant status, acculturation, ethnic identity, ethnic pride, and resilience [12,79,82,83]. At the very least, these findings underscore the importance of accounting for the health effects of such correlates when rigorously assessing the relationship between discrimination and health outcomes.

Despite these empirical advances, most studies do not disaggregate the Latino panethnic category into distinct ethnic groups, nor do they account for scale dimensionality (e.g., subtle vs. blatant) of discrimination. Little is known about how different types of discrimination differ across Latino ethnic groups at the national level and it remains unclear which scaling approaches are most appropriate for Latinos of different Latino ethnic groups. There is a dearth of research on whether measurement error and item responses affect the relationship between discrimination and health among and between different Latino Ethnic groups. By testing Latino ethnic differences in discrimination with nationally representative data, and applying improved measurement and analytic strategies, the present study informs debates about the contemporary relevance of racism and discrimination and moves beyond the Black-and-White binary that currently dominates academic debates.

On the theoretical front, no theory has operationalized and conceptualized, through clear propositions and testable hypotheses, which Latino groups are most likely to experience discrimination. Nor does a theoretical framework specify how the strength of the relationship between discrimination and poor health outcomes varies across Latino subgroups. To date, there is little theoretical guidance on whether overt or subtle discrimination is more harmful and for which Latino groups. As a result, researchers lack theoretical clarity on whether discrimination exerts stronger effects on the health of some Latino groups than others, which type of discrimination is most harmful, and why such differences should be expected. The knowledge base on the relationship between discrimination and health is still in its theoretical infancy and struggling to move away from risk factor epidemiology. Still, insights from scholars of race relations suggest the importance of distinguishing between blatant and subtle forms of discrimination, offering a potential foundation for advancing this inquiry.

Racial and ethnic stratification scholars have made the case for distinguishing between subtle versus blatant forms of discrimination. For instance, Latinos living in large Northeastern metropolitan areas may encounter more subtle forms of discrimination, given that the U.S. Civil War and subsequent civil rights legislation rendered blatant acts of de jure discrimination both illegal and culturally taboo in the North [84,85]. Pettigrew and Meertens (1995) define blatant prejudice as overt, hostile rejection of out-groups, in contrast to subtle prejudice, which takes the form of indirect, socially acceptable expressions of bias [85]. Their cross-national survey evidence demonstrates that once explicit racism becomes taboo, it often shifts underground into coded, symbolic attitudes. Massey and Denton (1993) further document how segregation in the South was enforced through de jure Jim Crow legal statutes, producing overt discrimination in schooling, housing, and public accommodations [84]. In contrast, Northern states abolished formal segregation earlier and enacted stronger civil rights protections against discrimination. As a result, Southern Whites, shielded by Jim Crow laws, could openly express hostile racial beliefs well into the mid-twentieth century, whereas Northern Whites faced stronger legal and cultural pressures to suppress overt discrimination. In the North, expanding civil rights activism and the failed attempt at integrated institutions reinforced norms against explicit discrimination, creating a cultural expectation of silence (“not saying out loud what you think”). By contrast, in the South these norms emerged later and only partially, leaving overt slurs and exclusionary practices far more commonplace.

The literature on colorism provides some further insights about the need to differentiate between subtle and blatant forms of discrimination. This literature demonstrates that skin tone and phenotype deepen racial and ethnic stratification by privileging lighter-skinned people of color and marginalizing darker-skinned, Black, and Indigenous individuals, including Latinos [75,86–88]. These dynamics significantly constrain the life chances of people of darker complexion and Black phenotype when compared with the privileges afforded to Whites, as POC experience more frequent and severe discrimination than their White counterparts. Importantly, such inequalities are often obscured and legitimized through the persistence of color-blind ideology, which incorrectly frames race, color, and discrimination as issues of social class and culture or characteristics of the past and a bygone era. Bonilla-Silva (2021) argues that color-blind ideology sustains racial and ethnic hierarchies through unspoken and subtle forms of discrimination [4].

Framed by this broader historical context, distinguishing between blatant and subtle forms of discrimination is crucial, as each exerts unique influences on individual experiences and social dynamics. While overt or blatant discrimination is easier to detect as it continues to directly shape the lives of POC, covert or subtle practices such as microaggressions endure under the veneer of color-blindness and may be more difficult to perceive. As Stanke et al. (2024) argue, subtle prejudice is perceived as more socially acceptable and as less overtly discriminatory than blatant prejudice, even though subtle prejudice is reported more frequently [89]. These subtle expressions—such as exaggerating cultural differences or making coded remarks—also elicit negative emotional reactions and are harder to identify as discriminatory. The authors argue that this social acceptability masks the harm of subtle prejudice, making it more difficult to challenge or even recognize.

This distinction between subtle and blatant types of discrimination has serious implications for measurement. Traditional discrimination scales may underreport subtle bias, and interventions may overlook its cumulative psychological toll. Capturing subtle forms is crucial because they persist in environments where overt discrimination is taboo, and they contribute to chronic stress, identity threat, and diminished well-being—especially among racially and ethnically minoritized populations. Thus, parsing these dimensions of discrimination allows researchers to capture how explicit policies and coded practices operate together to reinforce racial and ethnic hierarchies, including racial and ethnic disparities in health. Without attention to both forms of discrimination, scholarship and policy risk underestimating the full spectrum of systemic racism and its consequences.

### 1.5. Propositions, Research Questions and Hypothesis

Building on these theoretical and empirical foundations, we put forward four propositions and associated research questions regarding discrimination and health outcomes in Latino populations. First, perceived discrimination is detrimental to health across these communities. Second, the health impacts of blatant and subtle discrimination differ in both magnitude and frequency. Third, the frequency and intensity of discrimination vary between Latino ethnic subgroups, contributing to heterogeneous health disparities. Finally, employing structural equation modeling techniques that account for scale dimensionality, item responses, and measurement error will provide a more accurate and nuanced understanding of how discrimination affects health across diverse Latino populations than the alpha scaling approach or the single discrimination question approach.

The following research questions thus prompt the current analysis:

1. Are there sub-ethnic differences in reports of perceived discrimination between Latino groups?
2. What is the factor structure of the EDS for all Latinos, and for Cubans, Mexicans, Puerto Ricans, and Latinos of other ethnic backgrounds?
3. Does the relationship between discrimination and health (i.e., concurrent validity) depend on the scaling approach that is used?
4. Specifically, what are the implications of using a composite scale approach compared to adopting a latent variable approach that accounts for measurement error, scale dimensionality, and skewed dichotomous factor indicators?

Following the lead of Guyll et al. (2001) [16], Barnes et al. (2004) [15], and Noh et al. (2007) [17] it is hypothesized that the EDS developed by Yan and colleagues (1997) [11] will have two dimensions (Subtle Mistreatment and Blatant Mistreatment) among all Latino groups, including Cubans, Mexicans, and Puerto Ricans. This study also hypothesizes that these two dimensions can be captured by a second order factor of perceived discrimination (see Figure 1) that can potentially be a stronger predictor of poor health than the other scaling approaches.

## 2. Materials and Methods

We used data from the 2002–2003 National Latino and Asian American Study (NLAAS), a nationally representative survey of the non-institutionalized Latino and Asian adult populations in the U.S. The data collection was funded primarily by the National Institutes of Mental Health and is part of the Collaborative Psychiatric Epidemiology Surveys (CPES). As detailed by Heeringa et al. (2004) [90], NLAAS used a four-stage national probability sample design with oversamples of adults from select ethnic origins (e.g., Cuban and Puerto Rican) to increase sample sizes that allow for examining interethnic group differences. Data were collected through face-to-face interviews in English or Spanish in the participant’s home unless a telephone interview was requested. More detailed information on the overall design, sampling procedures, and survey instruments of NLAAS has been previously described by CPES [91]. Because the NLAAS employed a multistage probability sampling design with differential selection probabilities, all analyses incorporated the provided sampling weights, strata, and primary sampling units (PSUs). We applied weights at the measurement stage (CFA and IRT models of the EDS) to ensure that factor loadings, thresholds, and fit indices reflect population-level estimates rather than unweighted sample characteristics. Failure to incorporate these design features at the CFA measurement stage can yield biased parameter estimates, underestimated standard errors, and inflated model fit [92]. By incorporating weights and design variables directly into the measurement models, we improve the accuracy and generalizability of our assessment of discrimination across Latino subgroups.

The NLAAS complex sample design yielded a sample of 2554 Latinos aged 18 or older, including 577 Cubans, 495 Puerto Ricans, 868 Mexicans, and 614 Latinos from other ethnic backgrounds (hereafter, other Latinos). The overall response rate for the Latino sample is 75.5%, which is excellent given that Latinos (especially lower-income Latinos) are more likely to reside in major urban areas—areas that tend to have lower response rates. Following the recommendations of Heeringa et al. (2024) [90] and Berglund (2008) [91], we adjusted the analyses for clustering (seclustr), stratification (sestrat), and Latino sample weight (nlswlat), with original NLAAS data variable names appearing in parenthesis. We also adjusted for Taylor linearized variance estimation and incorporated the subpopulation options so as not to compromise the sample size which can impact the degrees of freedom and levels of significance.

### 2.1. Measures

#### 2.1.1. Dependent Variables

We used two dependent variables—depression and chronic health conditions—to test the basic concurrent validity of the Everyday Discrimination Scale. As Landrine and Klonoff (1996) pointed out, one of the ways in which the concurrent validity of a scale is tested is by examining the relationship between a stress scale and psychiatric symptoms that the researcher expects to be related to a construct [93]. Given that most studies on discrimination focus on mental health outcomes [94], and that a central tenet of our argument is that discrimination is related to many other stratification outcomes [95,96], we include a measure of chronic health conditions, as it is also a variable of high interest in discrimination literature.

Depression is measured with ten questions from the Center for Epidemiological Studies Depression Scale (CES-D), which is one of the most widely used and validated screening scales of depression [97,98]. This study measures depression as a latent factor with 10 observed categorical indicators. Respondents were asked if, most days, they (1) had a small appetite, (2) talked/moved more slowly than usual, (3) had slowed or mixed up thoughts, (4) had unusual indecisiveness, (5) lost self-confidence, (6) felt not as good as others, (7) felt guilty, (8) felt irritable/grouchy/moody, (9) felt nervous or anxious, and (10) felt sudden attacks of intense fear or panic (α = 0.94). All 10 items loaded on one latent variable and had an excellent fit, even without correlated measurement errors (χ^2^ = 91.29, *p* < 0.01; RMSEA = 0.02, TL = 0.99). An almost identical fit was observed for Cubans, Mexicans, Puerto Ricans, and other Latinos. This latent depression variable had both form invariance and measurement invariance across Latino groups which are “necessary conditions for meaningful and accurate comparison of groups on construct(s) of interest” [99].

Because responses to these questions on depression are dichotomous (1 = yes, 0 = no), they were specified as categorical, and model fit was assessed with a weighted least squares estimator. Springer and Hauser (2006) argue that this is one of the best ways to model non-normal data, particularly when the number of categories is small, and the data do not approximate a normal distribution [100]. In the case of binary indicators that are highly skewed, such as these depression items (or highly skewed ordinal indicators, such as those found in the EDS, see below), treating such items as continuous can produce attenuated correlations between factor indicators [101], inflate chi-squared values [102], and result in the underestimation of error variances, standard errors, and factor loadings [103]. Therefore, it is important to correct these issues at the measurement stage to minimize the biases that may be introduced in the structural models. To the best of our knowledge, studies of Latino health have not made these important measurement corrections.

The second dependent variable is chronic health conditions, an outcome of interest in the discrimination and health literature [104,105]. Respondents reported if they ever had arthritis/rheumatism, back/neck problems, frequent or severe headaches, chronic pains, seasonal allergies like hay fever, a stroke, heart disease, heart problems, high blood pressure, asthma, chronic lung disease, diabetes/high blood sugar, stomach/intestinal ulcer, epilepsy, or cancer. We created a dichotomous variable (0 = none; 1 = one or more), indicating whether the respondent reported any of the chronic health conditions listed versus not having any of them. Other researchers have created a count variable from these conditions by summing the number of yes responses [106] or have relied on regression techniques such as zero-inflated regression models to model the high proportion of zeroes in this outcome [107]. In this study, chronic health conditions were dichotomized rather than treated as a count variable. We recognize that dichotomization reduces granularity and may obscure variation in severity or number of conditions. However, this approach was necessary to ensure adequate cell sizes for subgroup analyses across Latino ethnic groups. Given the relatively small sample sizes in certain subgroups, a count variable would have produced sparse cells. Our use of dichotomization thus represents a trade-off: it facilitates more reliable comparisons across groups but at the cost of some precision [108–110].

#### 2.1.2. Independent Variables

Following the lead of Williams et al. (1997), the EDS is used in the context of unfair treatment rather than in the context of race [111]. Respondents were asked nine questions about how often, in their day-to-day life, they were treated with less courtesy and respect than others, received poor service at restaurants/stores, were treated as not smart, people were afraid of them, were thought of as dishonest, people acted as if they were better than them, were called names and insulted, and threatened or harassed. Answers were coded to range from low to high discrimination (1 = never, 2 = less than once a year, 3 = few times a year, 4 = few times a month). These items had a high level of internal consistency in the Latino sample (α = 0.91), a finding similar to those reported by Pérez et al. (2008) with the NLAAS data [112]. According to Williams and Mohammed (2009) this is one of the most widely used measures of perceived discrimination given that it has several attractive features, including its brevity and its utility with minorities of different racial/ethnic backgrounds in the U.S. and elsewhere [40], including with Portuguese individuals [55], German adults [113], indigenous adolescents, [114] and adolescents in Spain [115], among other samples. Across studies, this scale is shown to have high reliability and internal consistency [116–120].

#### 2.1.3. Background Variables

To examine the robustness (i.e., concurrent validity) of the relationship between discrimination scales and health across Latino subgroups (i.e., Cuban, Mexican, Puerto Rican, and other Latinos), the multivariate models are adjusted for other important correlates of health. Following the lead of Park et al. (2018) [121], Pérez et al. (2009) [122], Lee and Ferraro (2009) [123], and the coding scheme of Pérez et al. (2008) [112], multivariate models are adjusted for the effects of background variables, including age, gender, marital status, education, household income, employment status, proficiency in the English language (ability to speak, read, and write), nativity (i.e., first, second, third generation), and strength of ethnic identity (i.e., salience of ethnic group). These are all correlates of health and including these variables in our multivariate models allows us to more accurately assess the strength of the relationship between discrimination and health. Table 1 below shows descriptive statistics across the variables by Latino ethnicity.

#### 2.1.4. Analytic Plan

Our modeling strategy begins by presenting descriptive statistics for all dependent and independent variables in Table 1. Descriptive statistics were estimated using Stata 17 [124], and mean differences in all variables by ethnic group were assessed using Wald Tests [125,126]. In Tables 2 and 3, we examine the model fit of different scaling approaches of discrimination using Confirmatory Factor Analyses (CFA) and Structural Equation Modeling (SEM) using Mplus 8.11 [127].

In Table 2, scale fit is evaluated when the nine items of the EDS are allowed to load on a single factor (models 1 and 2 for each ethnic group), on two first-order factors called subtle and blatant discrimination (models 3 and 4), and on a second-order factor of perceived discrimination with two first-order dimensions (models 5 and 6), as also captured in Figure 1. For ease of interpretation, we highlight the best-fitting model in bold letters. Our initial exploratory analyses (EFA) showed that the nine items of the EDS loaded on two distinct factors with the two-factor solution showing a better fit than the one-factor solution for all ethnic groups. In the CFA that appears in Table 2, the fit of each of these scaling approaches is evaluated for scales with and without measurement error. In addition, the fit of the EDS is also assessed by treating the factor indicators as continuous, and as ordered-categorical, in accordance with Item Response Theory. Because the EDS consists of ordinal Likert-type items, we used categorical confirmatory factor analysis (CFA) with a weighted least squares mean and variance (WLSMV) estimator. Treating factor loadings as continuous with maximum likelihood (ML) estimation (a common CFA approach in this literature) can inflate fit indices (i.e., falsely indicate good fit) and obscure multidimensionality. In contrast, categorical CFA/IRT parameterization yields more accurate estimates of discrimination’s dimensional structure and fit. In Table 2, the analyses are repeated separately for Cubans (models C-1 to C-6), Mexicans (M-1 to M-6), Puerto Ricans (P-1 to P-6), and Latinos of other backgrounds (O-1 to O-6).

Since the chi-squared statistic of overall model fit is sensitive to sample size and often leads researchers to reject good fitting models as sample size increases [128], model fit was gauged with additional indices, including Bentler’s (1990) Comparative Fit Index (CFI) with values greater than 0.95 representing a good fitting model [129]; the Root Mean Square Error of Approximation (RMSEA) index, with values greater than 0.10 indicating a poor fit and values of less than 0.05 indicating a good fit [130,131]; and the Bayesian Information Criterion (BIC), with smaller numbers representing better model fit [132]. Springer and Hauser (2006) suggest that, when comparing models, a difference of ten or more points in the BIC statistic indicates a better-fitting model [100]. The BIC statistic has the added advantage of being used to compare non-nested models.

SEM is a less biased technique for assessing the reliability and validity of latent variables compared to alpha scaling and has three major advantages over traditional multivariate techniques in that it allows for: (1) the explicit assessment of measurement error, (2) the estimation of latent (unobserved) variables via observed variables, and (3) testing the fit of model structure. We can also test if the parametrization of latent factor indicators as ordinal on the EDS produces a better fit than if the indicators are assumed to be continuous. As described above, these programs (Stata and Mplus) adjust standard errors for sampling stratification, clustering of individuals, unequal probability of selection, and allow for the analysis of subpopulations (e.g., Mexican, Cubans) with complex survey data [133] as necessitated by the NLAAS sampling design.

In Tables 3 and 4, we focus on the multivariate analysis and assess which type of discrimination scale (i.e., alpha, one-dimension, two-dimensions, second order) has the strongest relationship (appearing in bold font) with chronic physical health conditions and depression, respectively. These two tables control for the effects of other co-variates described in Table 1. In Table 5, our final table, we conduct a sensitivity analysis by comparing the separate and independent effects that each subscale of perceived discrimination (subtle vs. blatant) has on chronic health problems and depression by Latino ethnic group, also controlling for all other variables.

## 3. Results

### 3.1. Descriptive Statistics

Table 1 includes weighted descriptive statistics for the full Latino sample and by Latino subgroups. Overall, Puerto Ricans report higher symptoms of depression (=0.18) than Mexicans (x^−^ = 0.13) and other Latinos (x^−^ = 0.13). Puerto Ricans are also more likely to report having a chronic physical condition (68%) relative to Mexicans (56%) and other Latinos (58%).

Significant Latino subgroup differences in reports of discrimination also emerged. Notably, Cubans were less likely than Mexicans, Puerto Ricans, and other Latinos to report being treated with less courtesy, less respect, receiving poor service in stores/restaurants, being treated as not being smart, having people being afraid of them, to be treated as being dishonest, to have people behave as if they were better than them, to be insulted, and to be harassed. Compared to Mexicans, Puerto Ricans were significantly more likely to report being treated with less courtesy (x^−^ = 2.47 vs. 2.17), to be treated with less respect (x^−^ = 2.25 vs. 2.03), to receive poor service (x^−^ = 2.08 vs. 1.89), to report that others think they are not smart (x^−^ = 2.22 vs. 1.99), to claim that others are afraid of them (x^−^ = 1.95 vs. 1.64), to be perceived as being dishonest (x^−^ = 1.82 vs. 1.59), to have people behave as if they were better than the respondent (x^−^ = 2.09 vs. 1.88), and to be harassed and/or threatened (x^−^ = 1.53 vs. 1.34). There were no significant differences observed in reports of discrimination between Mexicans and other Latinos. Overall, Puerto Ricans report the highest levels of discrimination and Cubans experience the lowest levels. We also find ethnic variation in marital status, education, household income, English language proficiency, generational status, and ethnic identity.

### 3.2. Confirmatory Factor Analysis

In Table 2, the factor structure of the EDS is explored for the full Latino sample and by Latino subgroups. In Panel A, model L-1, the single-factor model, fits the data poorly (RMSEA = 0.09, CFI = 0.87). This poor fit is observed regardless of whether factor indicators are parameterized as either continuous or ordered-categorical (RMSEA = 0.12, CFI = 0.97. The poor fit of this one-factor solution with no correlated measurement errors is also observed for Cubans (C-1: RMSEA = 0.10, CFI = 0.79), Mexicans (M-1: RMSEA = 0.11, CFI = 0.88), Puerto Ricans (P-1: RMSEA = 0.11, CFI = 0.83), and Latinos from other ethnic backgrounds (O-1: RMSEA = 0.09, CFI = 0.85). When the factor indicators are specified as ordinal-categorical, this one-factor solution without correlated errors still fits the data poorly for each of these ethnic groups. Notice that while the RMSEA index suggests a poor-fitting model for each Latino group (i.e., RMSEA > 0.10), the CFI reveals a marginally good fit for the one-factor solution when factor indicators are specified as categorical.

The CFI is above the cut-off point (0.95) for Cubans (C-1: CFI = 0.95), Mexicans (M-1: CFI = 0.97), Puerto Ricans (P-1: CFI = 0.95), and other Latinos (O-1: CFI = 0.95). However, this discrepancy in model fit between the RMSEA and the CFI is expected. The CFI is sensitive to non-centrality in the data and is also negatively affected when factor correlations approach zero [134,135], such as is the case with these categorical factor indicators.

Taken as a whole, the one-factor solution for the EDS with no correlated errors does not fit the data well. This suggests that reliance on alpha scales, which are assumed to be unidimensional and error-free, may not be the analytically optimal way to measure perceived discrimination in these Latino groups. Unfortunately, the error-free unidimensional scaling approach is widely adopted in studies of discrimination and Latino health.

The restriction of uncorrelated measurement error is relaxed in models L-2 (for the combined Latino sample in Panel A) of Table 2, and also for each respective ethnic group in models C-2 to O-2 (Panels B to E) of Table 2. In this second set of models, the error terms of the following items are allowed to correlate: courtesy with respect, afraid with harassed, and insult with harassed (see Figure 1). The dashed lines in Figure 1 show that, for Cubans, two additional parameters were freed to achieve a good fit: errors with respect to service, and afraid of dishonesty. We used Mplus-based modification indices to guide our decision about which errors to correlate and kept such correlations to a minimum to avoid over-fitting and saturating the model. Even with this basic adjustment, a significant improvement in model fit is observed. For the combined Latino sample with continuous factor indicators, model L-2, Panel A in Table 2 fits the data well (RMSEA = 0.044, CFI = 0.973).

We also tested the single-factor model (L2) with correlated errors for group invariance (i.e., configural, scalar, metric) by language of interview and found support for group invariance. This means that the latent factor fits well for both Spanish speakers and English speakers. For English respondents, χ^2^(24) = 180.72, *p* < 0.001; RMSEA = 0.080 (90% CI: 0.069–0.091); CFI = 0.985; TLI = 0.977; SRMR = 0.036. For Spanish respondents, χ^2^(24) = 135.99, *p* < 0.001; RMSEA = 0.057 (90% CI: 0.048–0.067); CFI = 0.993; TLI = 0.990; SRMR = 0.022. Given the excellent CFI/TLI and small SRMRs in both groups, the factor structure (i.e., configural invariance) was judged sufficiently similar. This per-group evidence supports proceeding to formal multigroup invariance testing (metric and scalar), because configural plausibility is established in both groups. Constraining factor loadings across groups produced only a small decrement in fit (metric ΔCFI = −0.0036), supporting metric invariance. For scalar invariance, constraining intercepts in addition to loadings produced a slightly larger decrement (scalar ΔCFI = −0.0096), which is slightly below relative to the conventional cutoff (−0.01) and within an acceptable limit. The RMSEA and SRMR hardly change across models in our results, and absolute fit (CFI *≈* 0.95) remains good in the scalar model. This finding supports a pragmatic interpretation that scalar constraints do not produce a substantive degradation of fit; therefore, the model also passes the check for scalar invariance. The three correlated residuals that appear in Figure 1 were significant in both groups (standardized courtesy with respect *≈* 0.60; insult with harassed *≈* 0.42–0.56; afraid with harassed *≈* 0.13–0.14), suggesting local dependence between these items. These findings are not surprising considering that the EDS has been validated across countries and language groups.

The BIC statistic provides useful information in this case. Notice how the BIC drops by more than 1570 points in model L-2 (BIC = 46,474), when compared to model L-1 (BIC = 48,044); this finding suggests that model L-2, with corrections for measurement error, is a better-fitting model than L-1, which does not correct for measurement error. The second-order factor with correlated errors, L-6, also provides a good fit. Overall, based on the findings in Table 2 for the combined Latino sample in Panel A, the two-factor solution of subtle and blatant discrimination fits the best. The second-order factor L-6 model with correlated errors also fits well. Both the L-4 and L-6 models fit better than the single-factor model, L-1.

However, the subgroup analyses in Table 2 show that even accounting for correlated error terms, the single-factor models 2 do not fit the data very well for Cubans (RMSEA = 0.059, CFI = 0.946), Mexicans (RMSEA = 0.058, CFI = 0.946), and Puerto Ricans (RMSEA = 0.064, CFI = 0.952). When the factor indicators are parameterized as ordered-categorical, the RMSEA fit index reveals a poor fit across all groups (RMSEA = 0.065) and for each of the other ethnic groups in Panels B to E. Thus, a simple correction for measurement error for the single-factor model does not produce a good-fitting model when ethnicity is considered. This finding also underscores the need to avoid the panethnic grouping of Latinos and the need to parameterize the EDS as having two dimensions, subtle and blatant discrimination.

Models 3 and 4 in each ethnic panel of Table 2 show the results for the two-factor solution (subtle discrimination and blatant discrimination) when the scale is measured without correction for measurement error (model 3), and when the adjustment is made for measurement error (model 4). Model 3 has a poor fit in the combined Latino sample regardless of whether factor indicators are specified as continuous (L-3: RMSEA = 0.071, CFI = 0.925) or as ordered-categorical (L-3: RMSEA = 0.095, CFI = 0.983). A similar poor fit in this two-factor solution without correction for measurement error is also observed for Cubans (C-3), Mexicans (M-3), Puerto Ricans (P-3) and other Latinos (O-3). Still, across all samples and for each type of parameterization, the two-dimensional model 3 fits better than the unidimensional model 1.

Model 4 reveals a markedly improved fit for the two-factor solution with correlated errors. The fit for the combined Latino sample is above the threshold for good fitting models for the combined Latino sample (L-4: RMSEA = 0.022, CFI = 0.994) as is the model fit for Cubans (C-4: RMSEA = 0.049, CFI = 0.96), Mexicans (M-4: RMSEA = 0.046, CFI = 0.993), Puerto Ricans (P-4: RMSEA = 0.044, CFI = 0.978), and other Latinos (O-4: RMSEA = 0.006, CFI = 0.999). A similar pattern of excellent model fit is observed when the factor indicators are parameterized as ordered-categorical for every ethnic group in this sample. In fact, compared to all models in Table 2, Model 4 has the overall better fit, including the lowest BIC statistic.

It appears that, at least from a measurement perspective, the EDS consists of two scales: subtle discrimination and blatant discrimination. This two-dimensional scale fits well for all Latinos and for Cubans, Mexicans, Puerto Ricans, and other Latino groups. The best fit is achieved when the discrimination variables are specified categorical factor indicators.

Models 5 and 6 in Table 2, which are fully captured in Figure 1, reveal a similar story. When the EDS is scaled as a second-order factor with two first-order dimensions, the model that corrects for measurement error (model 6) fits the data much better than the second-order model of perceived discrimination that does not correct for measurement error (model 5). Also, with continuous factor indicators, the second order factor with correlated measurement errors fits well for the combined Latino sample (L-6: RMSEA = 0.032, CFI = 0.986), Cubans (C-6: RMSEA = 0.055, CFI = 0.948), Mexicans, (M-6: RMSEA = 0.043, CFI = 0.984), Puerto Ricans (P-6: RMSEA = 0.057, CFI = 0.961), and other Latinos (O-6: RMSEA = 0.024, CFI = 0.992).

In contrast, the second-order factor with the categorical factor indicator does not fit well for any of the groups, as revealed by the RMSEA greater than 0.05. But notice how the CFI for a second-order factor with correlated indicators provides a marginally good fit. Still, for reasons that will become more apparent in the next section, where the concurrent validity of the EDS is examined, a second-order operationalization of the EDS may be the better scaling approach than the two-factor solution captured in model 4; this is especially the case when the structural relationship between discrimination and health is examined in a multivariate context, as shown next.

### 3.3. Concurrent Validity: Multivariate Relationship Between Discrimination and Health

In Tables 3 and 4, we test the concurrent validity of the EDS. In Table 3, the analysis turns to how each scaling approach affects the relationship between discrimination and chronic physical health problems, controlling for all other variables in Table 1. In Table 4, we explore how each scale affects the relationship between discrimination and depression. The analytic strategy is on how the alpha scaling approach compares to the other scaling approaches across ethnic groups. Overall, Table 3 shows that both the scaling approach and ethnic background matter. The results reveal that the composite scale (alpha scale) does not fit the data well and has a weaker relationship with health than scales that correct for measurement error and dimensionality. Although the differences between the alpha scale in column 2 and the last column of the second order factor with correlated errors are modest, they are not negligible.

Looking across the first row of coefficients for all Latinos in Table 3, the relationship between discrimination and chronic health is positive: those who experience discrimination in their daily lives more frequently have a greater likelihood of having a chronic physical condition than individuals with less frequent experiences with discrimination. As expected, the coefficient for the alpha scaling approach in Model 1 (β = 0.136, *p* < *0*.01) is significant and nearly identical in magnitude to the single latent-factor approach in Model 2 (β = 0.135, *p* < 0.01) that does not correct for measurement error. Notice, however, that the effect of discrimination for the single factor scale that corrects for measurement error in Model 3 is two percent stronger when compared to the alpha scale approach in Model 1 ([0.139 – 0.136]/0.139 = 0.02). This initial finding suggests that the alpha scaling approach tends to underestimate the relationship between discrimination and chronic physical health problems. A two percent difference might seem trivial, but it can make a difference in determining whether discrimination significantly harms health or not. Similarly, when we compare Model 1 to Model 7 in Table 3, the relationship between discrimination and chronic health conditions is four percent stronger ([0.143 – 0.136]/0.143 = 0.04) for all Latino groups. Table 3 also shows that for “Other Latinos”, the alpha scale does not reach statistical significance, whereas the second order factor with correlated errors is significant. Researchers who rely on the alpha scaling approach may conclude that the relationship between discrimination and chronic health conditions matters little for other Latino groups.

Models 4 and 5 in Table 3, for the combined Latino group, are based on the two-factor solution with and without correlated measurement error, respectively. Although Model 5 is the best-fitting model (see Table 2), neither the Subtle Mistreatment factor nor the Blatant Mistreatment factor is significantly related to chronic physical health when they are entered, together, into the regression equation in Models 4 and 5 of Table 3. This surprising lack of significance may occur because these two factors have a moderately high correlation (r > 0.65).

Although the variance inflated factor is less of 1.72, such a correlation between two independent variables inflates standard errors and deflates t-statistics. For researcher interested in exploring the effects of subtle and blatant effects of discrimination on health, there are two modeling options. Use the single factor latent variable with correlated errors and categorical indicators or use the second order factors with correlated errors and categorical indicators. The lack of significance between the separate subtle and blatant dimensions of discrimination and chronic health conditions is consistent with studies indicating that such as relationship is less robust than the relationship between discrimination and mental health outcomes [80]. To further explore this insight, we turn our attention enter the effects of subtle and blatant discrimination in the multivariate models separately, and we will return to this deeper analysis in Table 5 below.

Table 3 also shows how the alpha scaling approach underestimates the relationship between discrimination and chronic health in the other ethnic groups when compared to the second-order scale in Model 7. The bias is particularly pronounced among Cubans and Latinos from other backgrounds. Notice that the regression coefficient for Cubans in Model 1 (β = 0.144, *p* > 0.05) is statistically insignificant, and it is also 26 percent weaker when compared to the coefficient of the second-order factor in Model 7 (β = 0.195, *p* > 0.05) of Table 3. Had the analyses been based on the alpha scaling approach, the conclusion would have been that Cubans and other Latinos are not harmed by the indignities associated with this powerful stressor. Overall, the results in Table 3 reveal that the relationship between discrimination and chronic physical health problems is statistically significant and is best captured by measuring discrimination as a second-order factor.

Before discussing the effects of discrimination on depression in Table 4, it is worth digging a little deeper into the Chronic health problems. In Table 5, we first test the effects that each dimension of discrimination has on chronic health conditions and then depression. When two independent variables are correlated, they can be entered in the equation separately to address collinearity between subscales and isolate individual scale effects. In Table 5, where each subscale of discrimination is entered into the regression equation separately, both the subtle and blatant dimensions of perceived discrimination are significantly related to chronic health problems for all Latino groups. Whereas a standard deviation increase in subtle mistreatment increases the propensity of Latinos to have a chronic health problem by 0.137 standard deviations, the effect of blatant mistreatment is slightly weaker (β = 0.133, *p* < 0.01). Notice how the effects of subtle discrimination on chronic health conditions are stronger than the effects of blatant discrimination for all Latinos, Cubans, and Mexicans. In contrast, the effects of blatant discrimination on chronic health conditions a stronger for Puerto Ricans and other Latinos. This finding is consistent with those of Noh et al. (2007) for Korean Immigrants in Toronto, Canada [17], who found a stronger relationship between subtle discrimination and mental health than between blatant discrimination and mental health; it is also consistent with the work of race scholars who contend that subtle forms of discrimination take on added relevance today as display of more blatant forms of discrimination become less politically correct and publicly frowned upon [89,136,137]. Importantly, the fact that different types of discrimination affect chronic health in distinct ways across Latino ethnic groups highlights a critical issue: using a panethnic label such as “Latino” can obscure meaningful differences among subgroups.

Table 4 shows both similarities and key differences from the chronic health analyses in Table 3, now applied to depression. One similarity is that, for the combined Latino sample, the second-order scale in Model 7 (β = 0.246, *p* < 0.01) has a stronger relationship with depression than the alpha scale in Model 1 (β = 0.235, *p* > 0.01). Among Mexicans and Puerto Ricans, the second-order factor also has a stronger effect on depression than the alpha scale. For example, whereas the relationship between alpha discrimination and depression is not significant for Puerto Ricans in Model 1 (β = 0.107, *p* > 0.05), it gains statistical significance in Model 7 (β = 0.144, *p* < 0.05), highlighting the importance of the second-order factor scale and measurement error. A second similarity is that the single-factor model with correlated errors (Model 3) reveals a stronger relationship between discrimination and depression than the alpha scale in Model 1, for the combined Latino sample, Mexicans, and Puerto Ricans. Third, the two-factor solutions (Models 4 and 5) also show some surprising results, which, again, are typical with independent variables that are correlated. For example, there is a negative relationship between experiences with blatant discrimination and depression for Cubans, Puerto Ricans, and Other Latinos. But when these two scales of discrimination are entered into the model separately in Table 5, more experiences with subtle discrimination are related to depression. This finding corroborates those of Noh and colleagues [17]. However, blatant discrimination was associated with depression only among Mexican Americans in Table 5, which is probably due to the increase in anti-Mexican American sentiments that have been spreading and growing across the U.S. for some time. The one notable difference between the findings for depression in Table 4 and those for chronic health problems in Table 3 is the lack of a relationship between discrimination and depression among Cubans.

A second modeling alternative is to allow these two dimensions of subtle and blatant discrimination to load on a second-order factor of Perceived Discrimination, as shown in Figure 1 and in models 6 and 7 of Tables 3 and 4. Returning to Table 3, model 7 reveals a highly significant relationship between the second-order factor of perceived discrimination and chronic health problems: a one standard deviation increase in perceived discrimination increases the propensity of Latinos to have a chronic health condition by 0.143 standard deviations, holding all other variables in Table 1 constant. This relationship between discrimination and chronic health is four percent stronger in Model 7, when compared to the effect of the alpha scale in Model 1 (β = 0.136, *p* < 0.01). It appears, then, that in the combined Latino sample, the alpha scaling approach tends to underestimate the relationship between discrimination and chronic physical health problems. In Table 4, the second order factor also had the strongest effects on depression among All Latinos and Mexicans.

Overall, the findings suggest that alpha scaling is not the optimal modeling strategy since it either fails to capture significant relationship between discrimination and chronic health problems for Cubans and “Other Latinos”, and, also, alpha scaling fails to capture significant relationships between discrimination and depression for Cubans and Puerto Ricans. A better approach would be to use a single-factor latent scale of discrimination, but adjusting for correlated measurement error and treating factor indicators as ordinal under IRT. This would be the straightforward measurement approach. Still, the second order factor with two dimensions, subtle and blatant discrimination, and with correlated errors and categorical factor indicators captured some of the strongest and most statistically significant relationships between discrimination and chronic health problems, and between discrimination and depression. However, this second-order scaling approach did not have the best fit with the data, but with minimal to correlated measurement errors, the second-order scale fits well. The two factor solutions reveal some unexpected findings, which would be expected with collinear sub-scales. But it also reveals that scholars can look at the distinct effects of blatant and subtle discrimination on health outcomes, opening some important insights. We recommend researchers use either the one-factor solution or the second-order solutions, as just described.

## 4. Discussion

The results of this study reveal that perceived discrimination remains a pervasive and measurable determinant of Latino health. Participants reporting higher levels of discrimination also reported poorer mental and physical health outcomes—patterns that align with decades of research on the biopsychosocial consequences of racism. Yet to understand these findings fully, they must be located within the contemporary political environment in which race and belonging have been aggressively contested.

Over the past decade—and with renewed intensity during and after the Trump administration—the U.S. has witnessed an emboldened resurgence of White nationalist rhetoric and policies that explicitly target immigrant and minority populations. Anti-Latino immigration raids, family separations, and “zero tolerance” enforcement are not merely administrative decisions; they are the material expression of a broader White supremacist project that seeks to reassert racial hierarchy under the guise of national security and law and order. These structural aggressions generate what Hardeman et al. (2022) call “measurable mechanisms of structural racism”—institutionalized stressors that erode trust, undermine access to care, and reinforce health disparities long after a single policy is rescinded [138].

At the same time, the political backlash against Diversity, Equity, and Inclusion (DEI) programs and the ascendance of so-called “color-blind” ideology have undermined even modest gains in acknowledging and rectifying the effects of racism and discrimination as social determinants of poor health. The dismantling of DEI offices across universities, medical schools, and public agencies—often framed as a defense of “meritocracy” or “neutrality”—reasserts the racial status quo by erasing the institutional language needed to name inequity. This is not neutrality; it is the normalization of racial hierarchy behind the façade of fairness. It mirrors what Bonilla-Silva calls the “new racism,” in which discrimination is denied in principle but reproduced in practice through unabated and ignored discriminatory practices.

Under this logic, racism becomes less visible but more pervasive. Blatant bigotry gives way to a subtler regime of bias: microaggressions, dismissive provider interactions, assumptions about language or intelligence, and daily indignities that communicate exclusion without overt hostility. These encounters, which respondents in many studies describe as ordinary and recurring, illustrate how the erosion of DEI and the rise of color-blind rhetoric translate directly into lived experience. They produce what scholars describe as everyday racism—an accumulation of small acts that generate chronic stress, poor physical health, and psychological burden. Far from being isolated incidents, these experiences are patterned outcomes of structural conditions that legitimize bias while denying its existence.

Meanwhile, overt discrimination has not vanished; it has been re-legitimized. We see this in state sponsored violence by the Trump administration, as it unconstitutionally, without due process, and indiscriminately targets anyone who looks Latino, even citizens [139,140]. The same political forces that dismantle DEI also sanction open hostility toward Latinos through mass detention, racialized policing, and rhetoric that frames immigrants as threats, criminals, and even terrorists. These high visibility acts of discrimination embolden the more “polite” forms of exclusion that Latinos encounter in clinics, work-places, schools, and simply walking in their communities. Subtle and blatant discrimination therefore operate on a tandem, each reinforcing the other: the spectacle of public hostility makes private disrespect seem normal, while everyday slights create the social climate that allows overt discrimination to flourish.

Within healthcare and public-health research, these political and cultural dynamics shape how inequity is measured and interpreted. The retreat into individualistic explanations of health outcomes shifts responsibility from institutions to individuals, obscuring how macro-level racism manifests as micro-level discrimination. This narrow framing erases the everyday realities that participants in this study described—receiving poor service; being treated with less courtesy and respect; people assuming that they are not smart and dishonest; and people being afraid, insulting and harassing. Each experience may appear minor, but together they form a system of exclusion that produces measurable health consequences. In this way, the rollback of DEI and the entrenchment of color-blind ideology do not merely silence conversations about racism; they perpetuate the subtle and overt forms of discrimination that our data reveal.

As Burgos et al. (2017) argue in their usage of the “White racial frame” in Puerto Rican health research, the epistemological foundations of health-disparities scholarship themselves reflect racialized power relations [20]. When researchers abstract culture from policy and power, they risk reproducing the inequities they seek to remedy. The current climate—marked by anti-immigrant legislation, stigmatization of Latino identity, and the institutional silencing of antiracist discourse—renders such reflexivity urgent. Medical sociology and public health must maintain its objective and methodological rigor, but it cannot remain politically blind when politics itself has become a mechanism of exclusion [141].

The general recommendations for assessing the relationship between discrimination and health among Latinos that emerged from the present study were: (1) researchers should take great analytic care and not treat Latinos as one monolithic group that share identical experiences with discrimination; (2) scholars should move away from relying on single-item measures of discrimination as it may underestimate the relationship between discrimination and health; and (3) measurement error and scale dimensionality are important measurement issues that affect the fit of the EDS.

The primary objective of this paper was to examine the factor structure and concurrent validity of the widely used EDS and, in the process, address three gaps in the literature on discrimination stress and Latino health. First, little is known about ethnic differences with discrimination in the Latino population. The analyses in this paper began by examining if experiences with discrimination in the nine items of the EDS were similar between Cubans, Mexicans, Puerto Ricans, and Latinos of other backgrounds. Bivariate results showed statistically significant differences in each of the scale items, with Cubans reporting less frequent experiences with discrimination and Puerto Ricans reporting more daily experiences with discrimination than other groups. However, ethnic differences depended on the question and the group being compared. Thus, as Zsembik and Fennel (2005) note, “racial/ethnic comparative health research should avoid panethnic groupings, and explicitly acknowledge group distinctiveness”, given that such ethnic lumping tends to obscure ethnic heterogeneity that is so crucial for understanding both the causes, onset, and trajectory of health and well-being across ethnic lines [142].

These results also indicate that researchers should be cautious when relying on constructs of questionable validity and reliability, such as measures of discrimination that are based on a single question [41]. Single-item measures of discrimination might misestimate (e.g., attenuate) the relationship between discrimination and health outcomes, considering that the relationship between discrimination and health might be contingent on the type of question used. Therefore, it is vital to move away from relying on single-item measures of discrimination, mainly when the goal is to examine if discrimination still harms the well-being of racial/ethnic minorities. Unfortunately, this single-item measurement approach is one of two approaches adopted in discrimination and Latino health studies. At risk is nothing less than incorrectly concluding that discrimination does not harm the health of Latinos, when in fact, discrimination is one of the key drivers of health disparities in this population.

The second objective of this paper was to explore the factor structure of the EDS and evaluate how measurement error affects the fit of this scale. We also examined if specifying factor indicators as ordinal provides better results. To date, the dimensional structure of the EDS among Latinos of different ethnic backgrounds has been unknown, despite calls for research that evaluates the reliability and validity of perceived discrimination scales among minorities [38,143]. It remains unclear if the EDS is a one-dimensional construct in the Latino population, or if it has multiple dimensions, as has been reported in other studies with samples of African Americans and Korean Immigrants that have relied on the EDS [15–17].

The results of a confirmatory factor analysis (CFA) revealed that the nine items of EDS load on two scales: subtle and latent discrimination. The two-factor solution fit the data well in the combined Latino sample, as well as among Cubans, Mexicans, Puerto Ricans, and Latinos of other backgrounds separately. For each group, the two-factor solution fit the data particularly well when the latent factors were adjusted for correlated measurement errors among factor indicators. In addition, a second-order factor of perceived discrimination with two first-order dimensions of subtle and blatant mistreatment also had an acceptable fit across ethnic groups once the restriction of uncorrelated measurement error was relaxed. These findings indicate that the EDS is not a one-dimensional construct with no measurement error, as assumed by the composite scaling approach.

Measurement error is an important issue in any study of perceived discrimination, but it may be particularly problematic with Latino samples. For instance, Roth’s (2008) qualitative work has shown that many Latinos tend to deny the existence of discrimination and/or underreport such experiences when they are asked “yes/no” questions on whether they have ever experienced discrimination [144]. However, when respondents were probed further, even those who denied the existence of discrimination in their respective countries were able to describe, in rich detail, encounters they personally had with discrimination as well as the experiences of their friends and family members with this powerful stressor. These findings highlight the importance of considering both ethnic differences and the role that measurement error may play in survey scales of perceived discrimination. Thus, widely used composite alpha scales of perceived discrimination may not be appropriate since they mask differential item loadings across Latino ethnic groups and cannot be used to adjust for measurement error. Additionally, alpha scales that are based on a few yes/no survey questions may underestimate the effect of discrimination on health and the prevalence of these experiences. Therefore, researchers should use perceived discrimination scales that correct for measurement error.

The third issue that was explored in this paper was whether the relationship between discrimination and health (i.e., chronic physical health problems and depression) depends on the scaling approach that is used. Specifically, to what degree does the alpha scaling approach bias the relationship between discrimination and health, when compared to the effects of the two-factor scale (subtle and blatant), and, also, when compared to the second-order scale of perceived discrimination that accounts for measurement error (see Figure 1)? As expected, the relationship between discrimination and chronic physical health was attenuated by the composite scale approach. The alpha scale attenuation was particularly pronounced for Cubans and Latinos of other backgrounds to the extent that the relationship between discrimination and chronic physical health was not statistically significant. In contrast, the second-order scale of perceived discrimination with correlated measurement error revealed a statistically significant relationship between discrimination and chronic health (but not depression) for Cubans and other Latinos; those with more frequent experiences of discrimination had a greater likelihood of having a chronic health condition than individuals with less frequent experiences with discrimination. In general, the relationship between discrimination and these two health outcomes was stronger when the second-order factor was used, even when compared to the other scaling approaches.

The absence of a significant relationship between the second-order discrimination scale and depression among Cubans may stem from several factors. As shown in Table 1, Cubans reported the lowest levels of discrimination relative to other Latino groups, the highest levels of ethnic identity, and were more likely to be first-generation and/or foreign-born. Each of these has been documented as a protective factor. This null finding is consistent with the literature on the Hispanic Paradox, which shows that more recent and first-generation immigrants often have better health outcomes than later generations despite lower socioeconomic status [145]. It also aligns with research demonstrating that a strong ethnic identity can buffer the effects of stressors among Latinos [146]. In addition, Cubans benefit, relative to other Latino groups, from other protective resources, including higher levels of education, greater household income, lower poverty rates, and strong social support within ethnic communities, as discussed above. Still, Table 4 showed that experiences with subtle discrimination were harmful to the mental health of Cubans, but blatant forms actually decrease depression. Perhaps subtle forms are internalized, whereas blatant forms are viewed not as an attack on the individual but as an attack on the ethnic group.

An equally important finding is that when the two scales of subtle and blatant discrimination were simultaneously entered into a multivariate model, they misestimated the relationship between these two forms of discrimination and health, a classic case of collinearity. However, when these two subscales were entered separately into the models, subtle experiences with discrimination had a stronger health effect than experiences with blatant forms of discrimination, a finding that is consistent with the work of Noh et al. (2007), who argue that “subtle racial bias may provoke significant stress by creating ambiguities in terms of social identity [17].” The stronger relationship between subtle discrimination and health also reflects the notion that today, “discrimination is mostly subtle, apparently nonracial, and institutionalized”, and has been observed by scholars writing from overlapping theoretical vantage points, including ‘laissez-faire racism’, ‘symbolic racism’, ‘aversive racism’, and ‘color-blind racism’ [4]. Regardless of the reason for this finding, the modeling recommendation reached in the current study is for scholars to refrain from using either the single-item approach or the alpha scale approach.

The results of this study strongly indicate two alternative modeling approaches. The preferred model is to use a second-order scale of perceived discrimination with two first-order dimensions of subtle and blatant mistreatment (see Figure 1). The second approach is to examine the separate effects of subtle or blatant experiences with discrimination in a multivariate context. Perhaps most importantly, researchers need to correct for measurement error when examining the effect of perceived discrimination on health. Moreover, reliance on composite scales may not be the analytically optimal choice either, considering that the two assumptions of this scaling approach (i.e., unidimensionality and no correlated measurement errors) often do not hold in other studies that have examined the factor structure of various scales of perceived discrimination [10,93,147,148]. Forman (2003) argues that it is important to consider the multidimensional nature of discrimination since failing to do so “can underestimate the psychological impact of discrimination on African American well-being. This paper extends Forman’s insight into the Latino population [149].

Limitations and future research. The analyses in this paper rely on the 2002–2003 National Latino and Asian American Study (NLAAS). Because these data were collected more than two decades ago, important social and policy changes since then—heightened anti-immigrant enforcement and rhetoric, the rise of overt and coded racist political discourse in the 2010s, the COVID-19 pandemic, and more recent rollbacks and contestations of Diversity, Equity, and Inclusion efforts—may have altered the prevalence, expression, and health consequences of perceived discrimination among Latino groups. These temporal changes limit the direct generalizability of our prevalence estimates and may shift which EDS dimensions (subtle versus blatant) are most salient or harmful in contemporary settings. That said, NLAAS offers clear advantages for the measurement questions we address: a nationally representative sampling frame for Latino subgroups, oversamples of Cubans and Puerto Ricans that enable subgroup CFA/IRT analyses, and interviewer language options that permit valid cross-language measurement testing. Those features make NLAAS well-suited for testing factor structure and measurement-error corrections, even while acknowledging that absolute levels and some structural relations may evolve over time. Future research should therefore replicate and extend our findings using more recent, nationally representative datasets and cohort studies (for example, subsequent NLAAS waves or studies of HCHS/SOL, COVID-era population health surveys, and contemporary CPES-style samples) that capture post-2016 and post-2020 political and public-health contexts. Such work should, at a minimum, (1) re-evaluate EDS dimensionality and measurement invariance across language and ethnic subgroups; (2) test whether the relative contributions of subtle versus blatant discrimination to mental and physical health have shifted; and (3) examine whether structural-level exposures (local immigration enforcement, pandemic-related discrimination, anti-DEI policies) moderate individual-level discrimination–health associations. These replication and extension efforts will determine whether the second-order factor structure and the measurement recommendations we advance here remain robust under contemporary social conditions.

We acknowledge that intersectional characteristics such as skin tone, immigrant generation, and English proficiency likely shape how discrimination is perceived and how it affects health within Latino subgroups. Testing these moderators (and related mediated pathways) requires targeted theoretical development, interaction and moderated-mediation models, and sufficient subgroup sample sizes and statistical power. Future research can explore if blatant discrimination interacts with ethnic identity and other factors that appear in Table 4 to produce this negative relationship between discrimination and depression. While important, such mediating and moderating analyses are beyond the scope of the present measurement-focused paper. We therefore recommend that future research—ideally using larger or more recent datasets and designs explicitly powered for intersectional inference—examine how skin tone, generation, language proficiency, and related axes of stratification alter both (a) the dimensional salience of subtle versus blatant discrimination and (b) discrimination’s downstream impacts on mental and physical health. We plan a follow-up study that will implement these tests with the appropriate theoretical framing and statistical approach.

## 5. Conclusions

In short, the case has been made, on both empirical and theoretical grounds, that measures of perceived discrimination are inherently complex, and also that such experiences are multifaceted. The current analysis adds weight to our understanding of the complexity of discrimination. These findings suggest that the relationship between discrimination and health can be more clearly understood with more sophisticated analyses. Future research should further validate the discriminant, convergent, and criterion validity of the second-order model presented here with Latinos of different backgrounds and other minorities, such as Asian Americans. The second-order factor developed in this paper can be tested by examining its correlation with other indices of discrimination and other stressors, particularly daily hassles, and its criterion validity can be assessed by examining how this scale correlates with previously validated scales of perceived discrimination. In addition, does the EDS have the same structure in cases involving different types of discrimination, such as discrimination due to race, language, ethnicity, gender, and sexual orientation [150]? There is a need to test the fit and validity of this scale among Latinos of other ethnic backgrounds (e.g., Dominicans) and other Latinos of color who may be victims of both ethnic and color discrimination. Scholars can also examine the fit of the EDS between Latinos who identify as Black compared to Latinos who do not identify as Black. More broadly, these different scales can be used to examine if discrimination affects other stratification outcomes, not just among Latinos, but other racialized minorities as well.

The relationship between discrimination and the life chances of individuals is fundamental to the study of social stratification. Whether discrimination matters or does not matter in the study of racial and ethnic stratification is largely an empirical question. Instead of ignoring the role of discrimination or assuming (i.e., not testing) that discrimination is the underlying factor that drives racial and ethnic differences in life chances, the results in this study provide some valuable insights on how to measure discrimination in survey research. These scaling approaches promise to have widespread utility for stratification scholars interested in exploring the relationship between discrimination and other stratification outcomes. Because this is the first study to test the factorial structure and basic construct validity of the EDS among Latinos of various ethnicities, in the ways conducted above, several areas for exciting future research have been opened.

Taken together, these insights position the current study’s findings as both empirical evidence and moral indictment. The persistence of perceived discrimination among Latinos is not accidental; it reflects a society in which racialized policing, anti-immigrant enforcement, and attacks on DEI operate as interconnected technologies of racial control. A truly antiracist health agenda must therefore move beyond “cultural competency” to confront structural causality—the policies, institutions, and ideologies that make race matter in the distribution of health, as proposed by the Racialized Place Inequality Framework [20].

Future research should quantify not only the prevalence of discrimination but also the institutional practices and political discourses that sustain it. As Hardeman et al. (2022) emphasize, measuring structural racism is both a methodological and ethical necessity if health equity is to be achieved [138]. Likewise, as Burgos Rivera García (2017) [20] and Yearby et al. (2023) [141] remind us, this work is inseparable from the broader struggle against white supremacy and economic injustice. In a moment when color-blind ideology and anti-DEI politics threaten to erase decades of civil-rights progress, reaffirming the centrality of race, power, and history in Latino health research is not only a scientific responsibility but a civic imperative.

## Figures and Tables

**Table 1.** Descriptive and Bivariate Statistics for all Variables by Latino/a Ethnic Background, NLAAS 2002–2003.

	Cubans	Puerto Ricans	Mexicans	Other Latinos	All Latinos
	N = 577	N = 495	N = 868	N = 614	N = 2554
Depressive Symptoms					
Depressive Symptoms	0.153	**0.185** ^M, O^	0.133	0.13	1.146
Chronic Physical Health (1 = yes, 0 = no)	0.651^M^	**0.680** ^M, O^	0.564	0.589	0.587
Perceived Discrimination					
Courtesy	1.911^P, M, O^	**2.470** ^M^	2.176	2.272	2.29
Disrespect	1.761^P, M, O^	**2.225** ^M^	2.039	2.065	2.129
Service	1.647^P, M, O^	**2.083** ^M, O^	1.887	1.875	1.964
Not Smart	1.644^P, M, O^	**2.220** ^M^	1.99	2.013	2.101
Afraid	1.458^P, M, O^	**1.956** ^M, O^	1.641	1.822	1.807
Dishonest	1.404^P, M, O^	**1.821** ^M^	1.596	1.677	1.699
Better	1.557^P, M, O^	**2.092** ^M^	1.883	1.969	1.993
Insult	1.321^P, M, O^	**1.747**	1.594	1.608	1.677
Harassed	1.222^P, M, O^	**1.539** ^M^	1.343	1.414	1.436
Background Variables					
Age	**38.3**	37.9	37.4	37.9	37.6
Gender (1 = Male)	0.517	0.516	0.522	**0.523**	0.521
Marital Status					
Married/Cohabitating (unmarried)	0.608^M, O^	0.542^M^	**0.691** ^O^	0.575	0.639
Divorced	0.165	0.169	0.121^O^	**0.168**	0.141
Never Married	0.226	0.288^M,^	0.186^O^	0.256	0.218
Educational status					
High school or less	0.205^P, M, O^	0.316^M^	**0.522** ^O^	0.341	0.434
High school graduate	0.272	**0.305** ^M^	0.244	0.241	0.251
Some college	0.267^M^	0.266^M^	0.159^O^	**0.28**	0.21
College graduate or more	**0.254** ^P, M, O^	0.112^M^	0.072^O^	0.137	0.104
Household income					
Less than $15,000	0.218	0.248	0.296	0.235	**0.27**
$15,000–$35,000	0.237^M^	0.225^M^	**0.308**	0.261	0.283
$35–$75,000	0.26	0.298	0.259	0.313	0.279
$75,000 or more	**0.283** ^M^	0.227^M^	0.134	0.189	0.166
Employed	**0.67**	0.612	0.621	0.657	0.633
Unemployed	0.064	0.071	0.071	**0.086**	0.076
Not in labor force	0.265	**0.315**	0.307	0.255	0.291
English language proficiency (1 = Yes)	0.502^P^	0.657^M, O^	0.404^O^	0.518	0.467
Generational Status					
First Generation or Foreign Born	**0.761** ^P, M, O^	0.405^M, O^	0.563	0.608	0.57
Second Generation	0.232^P^	**0.369** ^M, O^	0.22	0.168	0.221
Third Generation	0.006^P, M, O^	**0.224**	0.215	0.222	0.208
Strong Ethnic Identity (1 = Yes)	**0.919** ^M, O^	0.88	0.86	0.854	0.863

Notes:

P Significantly different from Puerto Rican mean (*p* < 0.05);

M significantly different from Mexican mean (*p* < 0.05);

O significantly different from Other Latino mean (*p* < 0.05); standard errors are available upon request. The highest average/mean scores appear in bold font.

**Table 2.** Fit Indices of Perceived Discrimination for Latinos of Different Ethnic Backgrounds, NLAAS 2002–2003.

	Continuous Factor Indicators					Categorical Factor Indicators			
	χ2	DF	RMSEA	CFI	BIC	χ2	DF	RMSEA	CFI
L-1. Single Factor Model	561.149*	27	0.090	0.874	48,044.608	1128.780*	27	0.129	0.968
L-2. Single Factor Model w/correlated errors	139.758*	24	0.044	0.973	46,474.553	274.270*	24	0.065	0.993
L-3. Two Factor Model	344.856*	26	0.071	0.925	47,157.945	607.333*	26	0.095	0.983
**L-4. Two Factor Model w/correlated errors**	**49.477** *	**23**	**0.022**	**0.994**	**46,144.285**	**70.248** *	**23**	**0.029**	**0.999**
L-5. Second-order factor	369.194*	27	0.072	0.919	47,196.792	1427.927*	27	0.145	0.959
**L-6. Second-order factor w/correlated errors**	84.690*	24	0.032	0.986	46,250.991	1311.529*	24	0.148	0.963
C-1. Single Factor Model	182.617	27	0.101	0.794	9593.038	207.774*	27	0.109	0.952
C-2. Single Factor Model w/correlated errors	62.724	21	0.059	0.946	9077.138	65.808*	21	0.062	0.988
C-3. Two Factor Model	115.896	26	0.078	0.881	9278.403	138.964*	26	0.088	0.970
**C-4. Two Factor Model w/correlated errors**	**52.400**	**22**	**0.049**	**0.960**	**9020.822**	**50.916** *	**22**	**0.048**	**0.992**
C-5. Second-order factor	122.890	27	0.079	0.873	9293.591	292.358*	27	0.132	0.930
C-6. Second-order factor w/correlated errors	62.669	23	0.055	0.948	9048.521	231.752*	23	0.127	0.945
M-1. Single Factor Model	298.996	27	0.110	0.880	16,016.956	473.407*	27	0.141	0.973
M-2. Single Factor Model w/correlated errors	91.125	24	0.058	0.970	15,557.183	129.071*	24	0.073	0.994
M-3. Two Factor Model	190.680	26	0.087	0.928	15,750.638	250.542*	26	0.102	0.986
**M-4. Two Factor Model w/correlated errors**	**41.129**	**23**	**0.046**	**0.992**	**15,445.714**	**46.649** *	**23**	**0.035**	**0.999**
M-5. Second-order factor	204.299	27	0.089	0.922	15,762.364	606.527*	27	0.161	0.964
M-6. Second-order factor w/correlated errors	60.169	24	0.043	0.984	15,479.137	531.809*	24	0.159	0.969
P-1. Single Factor Model	187.105	27	0.112	0.830	9805.920	210.462*	27	0.120	0.956
P-2. Single Factor Model w/correlated errors	69.626	24	0.064	0.952	9523.171	100.542*	24	0.082	0.982
P-3. Two Factor Model	119.367	26	0.088	0.901	9658.646	138.299*	26	0.096	0.973
**P-4. Two Factor Model w/correlated errors**	**43.997**	**23**	**0.044**	**0.978**	**9473.655**	**51.050** *	**23**	**0.051**	**0.993**
P-5. Second-order factor	128.591	27	0.09	0.892	9671.849	237.946*	27	0.129	0.949
P-6. Second-order factor w/correlated errors	60.606	24	0.057	0.961	9505.863	206.941*	24	0.127	0.956
O-1. Single Factor Model	183.956	27	0.099	0.855	11,874.541	365.420*	27	0.146	0.954
O-2. Single Factor Model with correlated errors	45.425	24	0.039	0.980	11,399.613	104.958*	24	0.076	0.989
O-3. Two Factor Model	116.268	26	0.077	0.917	11,623.695	206.398*	26	0.108	0.976
**O-4. Two Factor Model w/correlated errors**	**23.565**	**23**	**0.006**	**0.999**	**11,327.920**	**40.410** *	**23**	**0.036**	**0.998**
O-5. Second-order factor	121.909*	27	0.077	0.912	11,629.806	405.408*	27	0.154	0.949
O-6. Second-order factor with correlated errors	32.499	24	0.024	0.992	11,350.686	384.518*	24	0.159	0.951

Notes:

* *p* < 0.05.

All fit indices are adjusted for complex survey design using Taylor Linearization. Fit indices for continuous factor indicators are produced with a robust maximum likelihood estimator; fit indices for categorical factor indicators are based on a robust weighted least squares estimator; the best-fitting model is highlighted in bold font.

**Table 3.** Standardized Logistic Regression Coefficients Comparing the Effects of Different Scales of Perceived Discrimination on Chronic Physical Health Problems by Latino Ethnic Group.

	Alpha Scale (Model 1)	Single Factor (Model 2)	Single Factor Model with Correlated Errors (Model 3)	Two Factor Model (Model 4)		Two Factor Model with Correlated Errors (Model 5)		Second Order Factor (Model 6)	Second Order Factor with Correlated Errors (Model 7)
				Subtle Mistreatment	Blatant Mistreatment	Subtle Mistreatment	Blatant Mistreatment		
All Latinos	0.136*** (0.046)	0.135*** (0.034)	0.139*** (0.042)	0.074 (0.082)	0.067 (0.094)	0.088 (0.155)	0.055 (0.153)	0.141*** (0.031)	0.143*** (0.032)
Cubans	0.144 (0.140)	0.128* (0.072)	0.131* (0.178)	0.104 (0.165)	0.060 (0.191)	0.113 (0.391)	0.020 (0.385)	0.134* (0.066)	0.195* (0.066)
Mexicans	0.148*** (0.058)	0.156*** (0.040)	0.161*** (0.082)	0.123 (0.143)	0.039 (0.178)	0.181 (0.252)	−0.017 (0.262)	0.165*** (0.038)	0.165*** (0.038)
Puerto Ricans	0.222** (0.125)	0.211*** (0.084)	0.220*** (0.106)	0.083 (0.189)	0.141 (0.245)	0.102 (0.371)	0.124 (0.378)	0.224*** (0.078)	0.225** (0.079)
Other Latinos	**0.125** (0.100)	0.108* (0.067)	0.109* (0.081)	−0.026 (0.172)	0.137 (0.179)	−0.069 (0.315)	0.179 (0.288)	0.113* (0.061)	0.113* (0.061)

Notes:

* *p* < 0.05;

** *p* < 0.01;

*** *p* < 0.001 (two-tailed tests).

Latent factor indicators are categorical and estimated with a robust maximum likelihood estimator. All regression coefficients in this table are fully standardized; standard errors appear in parentheses. Adjusted results control for the effects of all independent variables in Table 1. Standard deviation appears in parentheses. The strongest relationship appears in bold font.

**Table 4.** Standardized Logistic Regression Coefficients Comparing the Effects of Different Scales of Perceived Discrimination on Depression by Latino Ethnic Group.

	Alpha Scale (Model 1)	Single Factor (Model 2)	Single Factor Model with Correlated Errors (Model 3)	Two Factor Model (Model 4)		Two Factor Model with Correlated Errors (Model 5)		Second Order Factor (Model 6)	Second Order Factor with Correlated Errors (Model 7)
				Subtle Mistreatment	Blatant Mistreatment	Subtle Mistreatment	Blatant Mistreatment		
**All Latinos**	0.235*** (0.050)	0.234*** (0.035)	0.241*** (0.041)	0.221** (0.094)	0.025 (0.106)	0.301* (0.164)	−0.053 (0.160)	0.243*** (0.032)	0.246*** (0.032
**Cubans**	0.047 (0.151)	0.036 (0.059)	0.038 (0.066)	0.407*** (0.135)	−0.373** (0.148)	0.914** (0.441)	−0.878** (0.408)	0.037 (0.054)	0.041 (0.053)
**Mexicans**	0.268*** (0.079)	0.280*** (0.043)	0.286*** (0.050)	0.062 (0.112)	0.228** (0.116)	0.047 (0.196)	0.244 (0.184)	0.290*** (0.038)	0.293*** (0.038)
**Puerto Ricans**	0.107 (0.094)	0.110* (0.055)	0.110* (0.069)	0.239* (0.128)	−0.127 (0.173)	**0.350** (0.247)	−0.239 (0.262)	0.144* (0.052)	0.144* (0.052)
**Other Latinos**	0.268*** (0.110)	0.251*** (0.052)	0.258*** (0.060)	0.519*** (0.127)	−0.258** (0.140)	0.838*** (0.231)	−0.576** (0.226)	0.262*** (0.049)	0.265*** (0.049)

Notes:

* *p* < 0.05;

** *p* < 0.01;

*** *p* < 0.001 (two-tailed tests).

Latent factor indicators are categorical and estimated with a robust maximum likelihood estimator. All regression coefficients in this table are fully standardized; standard errors appear in parentheses. Results control for the effects of all independent variables in Table 1. Standard errors appear in parentheses. The strongest relationship appears in bold font.

**Table 5.** Comparing the Separate/Independent Effects of Each Subscale of Perceived Discrimination on Chronic Health Problems and Depression by Latino Ethnic Group.

	Chronic Health Problems		Depression	
	Subtle Mistreatment	Blatant Mistreatment	Subtle Mistreatment	Blatant Mistreatment
	Factor	Factor	Factor	Factor
**All Latinos**	0.137***	0.133***	0.253***	0.218***
	0.041	0.042	0.041	0.044
**Cubans**	0.129*	0.124	0.085	−0.023
	0.031	0.08	0.062	0.061
**Mexicans**	0.166***	0.146***	0.267***	0.286***
	0.045	0.059	0.055	0.052
**Puerto Ricans**	0.212***	0.218**	0.137**	0.076
	0.095	0.115	0.058	0.082
**Other Latinos**	0.09	0.117*	0.323***	0.176
	0.088	0.075	0.059	0.07

Notes:

* *p* < 0.05;

** *p* < 0.01;

*** *p* < 0.001 (two-tailed tests).

All regression coefficients are fully standardized; standard errors appear in parentheses. Results control for the effects of all independent variables in Table 1.

## Data Availability

The data are public available the Inter-University Consortium for Political and Social Science Research https://www.icpsr.umich.edu/web/ICPSR/studies/20240/datadocumentation.
